# Supplementary material for: Automated Parallel Dialysis for Purification of Polymers
Source: Polymers (Basel). 2022 Nov 10;14(22):4835. doi: 10.3390/polym14224835 (PMC9697721; doi:10.3390/polym14224835)
Supplement: Supplementary file 1 [file polymers-14-04835-s001.zip › polymers-1997666-supplementary.pdf]

Supplementary Information

Automated Parallel Dialysis for Purification of Polymers

İpek Terzioğlu [1,†], Carolina Ventura-Hunter [1,2], Jens Ulbrich [1], Enrique Saldívar-Guerra [2], Ulrich S. Schubert [1] and Carlos Guerrero-Sánchez [1,*]

1 Laboratory of Organic and Macromolecular Chemistry (IOMC), Friedrich Schiller University Jena, Humboldtstrasse 10, 07743 Jena, Germany
2 Polymerization Processes Department, Centro de Investigación en Química Aplicada (CIQA), Blvd. Enrique Reyna No. 140, Saltillo 25294, Coahuila, Mexico
* Correspondence: carlos.guerrero.sanchez@uni-jena.de
† Current address: Department of Polymer Science and Technology, Middle East Technical University, Çankaya, Ankara 06800, Turkey.

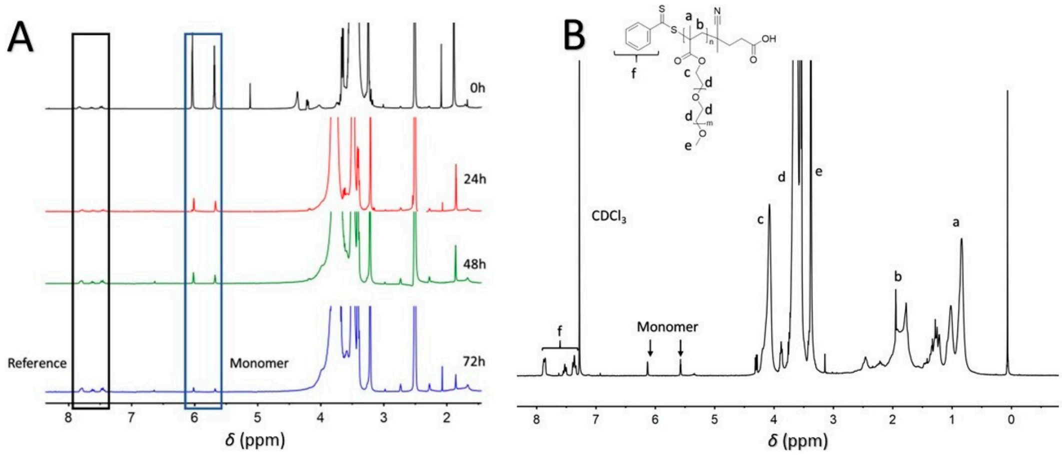

Figure S1. ^{1}H-NMR spectrum of P1. **A**) Dialysis of residual PEGMA, water suppression experiment in D_2O. **B**) P1 purified in $CDCl_3$.

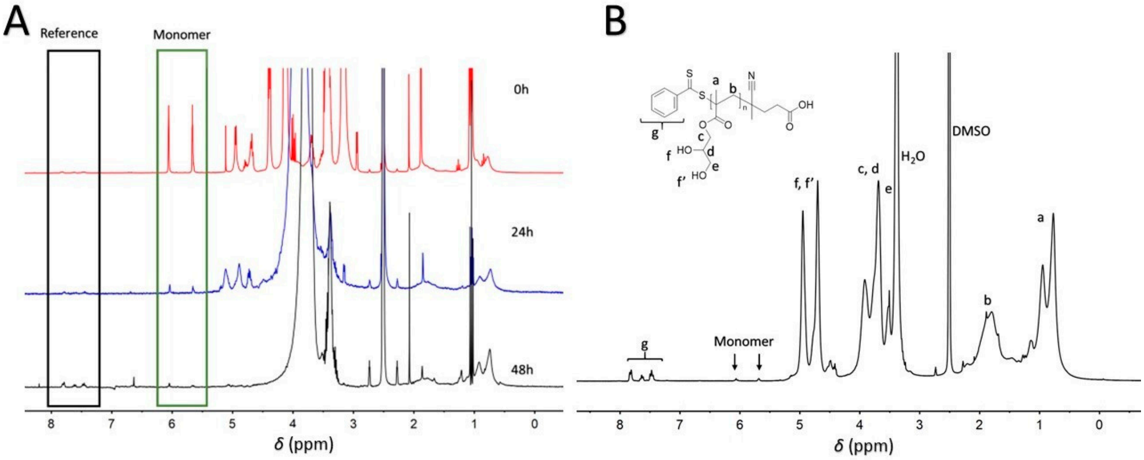

Figure S2. ^{1}H-NMR spectrum of P2. **A**) Dialysis of residual GMMA, water suppression experiment in D_2O. **B**) P2 purified in DMSO-*d*$_6$.

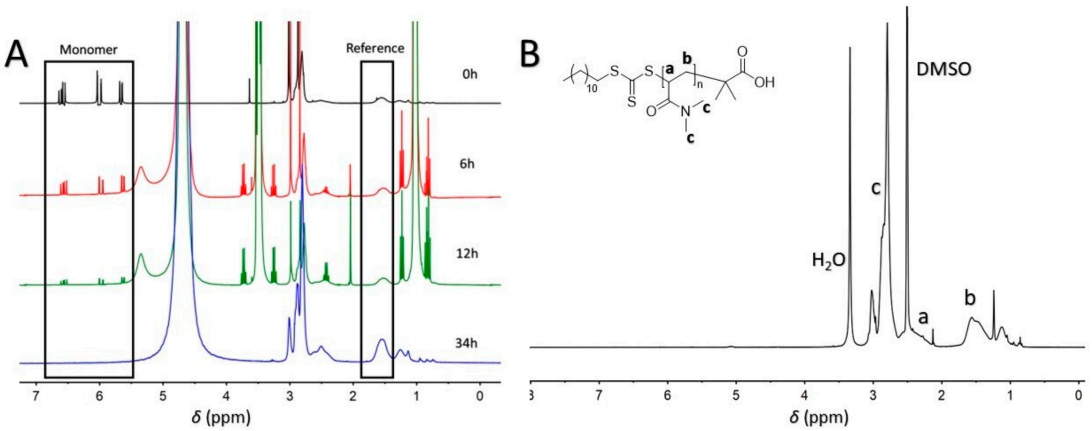

Figure S3. ^{1}H-NMR spectrum of P3. **A**) Dialysis of residual DMA, water suppression experiment in D_2O. **B**) P3 purified in in DMSO-d_6.

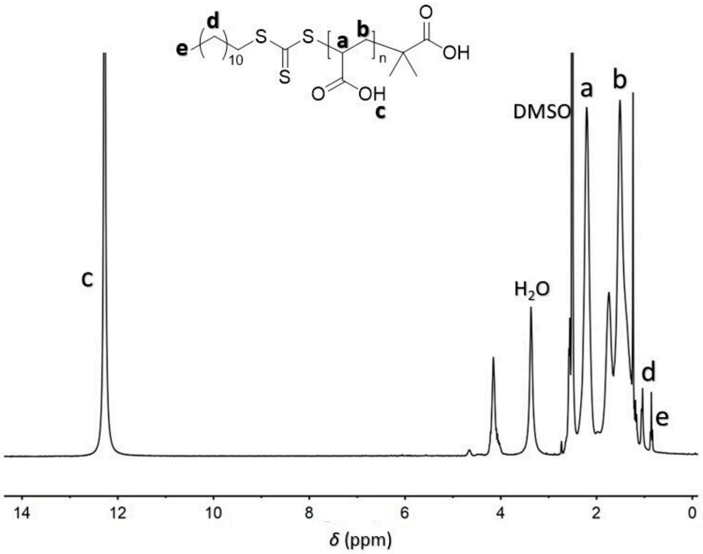

Figure S4. ^{1}H-NMR spectrum of P4 in DMSO-d_6.

Task	Name	Parameter	Description
1	Transfer Volumetrically	Transfer liquid from Reservoir 1 to Waste 1	Rinse needle, pumps and tubings
2	Transfer Volumetrically	Transfer liquid from R01 to D08	Transfer reaction solution into dialysis tubing
3	Set Timer	Set the timer Start	Start timer clock
4	Transfer Volumetrically	Transfer liquid from D08 to S01	Take sample from dialysis tubing into sample vial
5	Transfer Volumetrically	Transfer liquid from NMR Solvent to NMR vials	Add NMR Solvent to sample vial
6	Wait	Waiting for 6:00:00 hours after timer Start	Continue 6h after timer start
7	Transfer Volumetrically	Transfer liquid from Reservoir 1 to Waste 1	Rinse needle
8	Transfer Volumetrically	Transfer liquid from D08 to S02	Take sample from dialysis tubing into sample vial
9	Transfer Volumetrically	Transfer liquid from NMR Solvent to NMR vials	Add NMR Solvent to sample vial
10	Wait	Waiting for 12:00:00 hours after timer Start	Continue 12h after timer start
11	Transfer Volumetrically	Transfer liquid from Reservoir 1 to Waste 1	Rinse needle
12	Transfer Volumetrically	Transfer liquid from D08 to S03	Take sample from dialysis tubing into sample vial
13	Transfer Volumetrically	Transfer liquid from NMR Solvent to NMR vials	Add NMR Solvent to sample vial
14	Wait	Waiting for 18:00:00 hours after timer Start	Continue 18h after timer start
15	Transfer Volumetrically	Transfer liquid from Reservoir 1 to Waste 1	Rinse needle
16	Transfer Volumetrically	Transfer liquid from D08 to S04	Take sample from dialysis tubing into sample vial
17	Transfer Volumetrically	Transfer liquid from NMR Solvent to NMR vials	Add NMR Solvent to sample vial
18	Wait	Waiting for 24:00:00 hours after timer Start	Continue 24h after timer start
19	Transfer Volumetrically	Transfer liquid from Reservoir 1 to Waste 1	Rinse needle
20	Transfer Volumetrically	Transfer liquid from D08 to S05	Take sample from dialysis tubing into sample vial
21	Transfer Volumetrically	Transfer liquid from NMR Solvent to NMR vials	Add NMR Solvent to sample vial
22	Wait	Waiting for 36:00:00 hours after timer Start	Continue 36h after timer start
23	Transfer Volumetrically	Transfer liquid from Reservoir 1 to Waste 1	Rinse needle
24	Transfer Volumetrically	Transfer liquid from D08 to S06	Take sample from dialysis tubing into sample vial
25	Transfer Volumetrically	Transfer liquid from NMR Solvent to NMR vials	Add NMR Solvent to sample vial
26	Wait	Waiting for 48:00:00 hours after timer Start	Continue 48h after timer start
27	Transfer Volumetrically	Transfer liquid from Reservoir 1 to Waste 1	Rinse needle
28	Transfer Volumetrically	Transfer liquid from D08 to S07	Take sample from dialysis tubing into sample vial
29	Transfer Volumetrically	Transfer liquid from NMR Solvent to NMR vials	Add NMR Solvent to sample vial
30	Wait	Waiting for 72:00:00 hours after timer Start	Continue 72h after timer start
31	Transfer Volumetrically	Transfer liquid from Reservoir 1 to Waste 1	Rinse needle
32	Transfer Volumetrically	Transfer liquid from D08 to S08	Take sample from dialysis tubing into sample vial
33	Transfer Volumetrically	Transfer liquid from NMR Solvent to NMR vials	Add NMR Solvent to sample vial
34	Transfer Volumetrically	Transfer liquid from D08 to Chemical 4	Transfer dialysis liquid into vial

Figure S5. Prepared dialysis program in the software of Chemspeed ASW2000.
